# Experience with Ambulatory Management of Pleural Pathologies Utilizing Small-Bore Indwelling Pleural Catheters

**DOI:** 10.7759/cureus.1636

**Published:** 2017-09-01

**Authors:** Imad-ud-din Saqib, Mobeen Iqbal, Atif Rana, Saira Hassan

**Affiliations:** 1 Department of Plastic Surgery, Shifa International Hospital, Islamabad, Pakistan; 2 Department of Pulmonology & Critical Care, Shifa International Hospital, Islamabad, Pakistan; 3 Department of Interventional Radiology, Shifa International Hospital, Islamabad, Pakistan; 4 Hematology/oncology, Shifa International Hospital, Islamabad, Pakistan

**Keywords:** empyema, pleural disease, palliative care, systemic disease and lungs, malignancies, pneumothorax

## Abstract

Introduction

Pleural effusion is the excess fluid that accumulates in the pleural space. Pneumothorax is the collection of free air in the pleural cavity, while empyema is the collection of pus in the pleural cavity. Such pleural pathologies pose a great challenge to patients and health care professionals alike. While multiple management options exist, the major portion of it is carried out in the inpatient setting. We sought to evaluate the ambulatory use of indwelling pleural catheters for pleural pathologies, including malignant pleural effusion, empyema, and primary spontaneous pneumothorax.

Methods

We conducted a prospective case series analysis of 15 patients with various pleural pathologies in which an indwelling pleural catheter was placed by interventional radiologists on an outpatient basis and subsequently followed-up in a pulmonary clinic. Results were analyzed on the basis of clinical, as well as radiological progress with parameters being complete, partial, or no resolution. We also obtained prospective data on the quality of life of these patients.

Results

Six out of seven patients with malignant pleural effusion reported clinical (complete or partial) resolution, while three reported radiological (complete or partial) resolution. Two of the three patients with nonmalignant pleural effusions reported complete clinical as well as radiological resolution. All three patients with empyema reported complete clinical resolution and partial radiological resolution, while both patients with primary spontaneous pneumothorax reported complete clinical and radiological resolution. Patients reported preserved or improved quality of life with the whole process managed on an outpatient basis.

Conclusion

We report a high rate of clinical and radiological resolution in various pleural pathologies in our study, which is first of its kind from this part of the world. It demonstrates the feasibility of ambulatory management of pleural pathologies with a multidisciplinary approach.

## Introduction

Pleural pathologies are common in pulmonary practice. Malignant pleural effusions and pneumothoraces make up a significant number of patient visits to hospitals and hospital admissions worldwide [1].

The management of pleural effusion depends on the underlying etiology. Various management techniques have been reported and suggested by the recent, evidence-based, British Thoracic Society (BTS) guidelines. The options are broadly categorized as diagnostic and therapeutic in nature. Traditionally, effusions and pneumothoraces are aspirated by needle or drained through large bore chest tubes. Recurrent malignant effusions may require the instillation of a sclerosant, thoracic surgery (open or video-assisted), or placement of an indwelling pleural catheter. Similarly, ambulatory drainage with Heimlich valve, open thoracotomy with pleurectomy, video-assisted thoracoscopic surgery (VATS) with pleurectomy, pleural abrasion, and surgical chemical pleurodesis are used in cases of pneumothorax [2].

With the advent of small-bore catheters, better known as indwelling pleural catheters (IPC), management of pleural pathologies have changed significantly. IPCs are increasingly being used and multiple studies have shown no significant differences between the outcomes of large and small-bore chest tubes [3-5]. Many centers worldwide are now considering IPC as the first-line treatment in place of large-bore intercostal drains, with or without chemical pleurodesis for the management of pleural pathologies [6-7], and trials are underway to evaluate their efficacy, cost effectiveness, hospital stay, and quality of life [8-9].

IPCs are also being used in the management of tuberculous, parapneumonic, empyema, and transudative causes of pleural effusions [10].

In Pakistan, large-bore chest tubes are still the most frequently used intervention for drainage of pleural effusions and pneumothoraces with chemical or surgical pleurodesis [11-12]. IPC use is becoming popular in some of the tertiary care centers due to its ease and patient comfort.

Recent studies have described the use of IPC on an outpatient basis followed by subsequent outpatient follow-up in order to further decrease the length of hospital stay [13-15]. Our aim in this study is to describe our experience with outpatient insertion and management of IPCs, pleurodesis, the utilization of IPC, when required, and its effectiveness in relief of symptoms, patient ease, length of hospital stay, cost, and prognosis, along with any complications arising from it.

## Materials and methods

Patients and study design

We conducted a retrospective case series analysis from January 2015 to December 2016. All patients who had an IPC placed in the outpatient setting and remained in outpatient follow-up were included in the study. The data of the selected patients were collected from medical records. Patients with incomplete records or lost to follow-up were excluded from the study. An informed consent about the use of patient-related data was obtained prospectively from patients, and in cases where the patients were unable to give consent, consent was obtained from their relatives. The study was approved by the institutional review board of Shifa International Hospital, Islamabad, Pakistan.

Patient management

The patients were managed using a multidisciplinary approach involving physicians from the pulmonology, interventional radiology, and oncology divisions if required. The decision to insert the indwelling pleural catheter was based on the judgment of the pulmonologist. Patients were subsequently referred to the interventional radiologist for assessment and a final decision.

Before proceeding with the procedure, patients were required to have their complete blood count, prothrombin time, INR (international normalized ratio), and chest X-ray done. Every patient was told about the procedure, along with possible complications, and given an informed consent form to be signed by the patient or the next of kin according to hospital policy.

All patients undergoing IPC had same-day imaging prior to IPC insertion. Before the start of the procedure, patients' vitals, including temperature, blood pressure, and pulse, were observed for a few minutes to ensure clinical stability.

The patient was positioned in a sterile environment and landmarks were identified. For pneumothorax, the fourth or fifth intercostal space on the anterior axillary line was identified. In the case of pleural effusion or empyema, ultrasonography was used to identify free floating effusions or largest loculation and the landmark was decided on accordingly. The skin was anesthetized with 10 mL of 2% lidocaine. An 8-French or 10-French straight drain with multiple side-holes (Easydrain®, Vygon Co, Ecouen, France) or size 8-French to 12-French pigtail catheter was used according to the type of pleural pathology.

**Figure 1:**
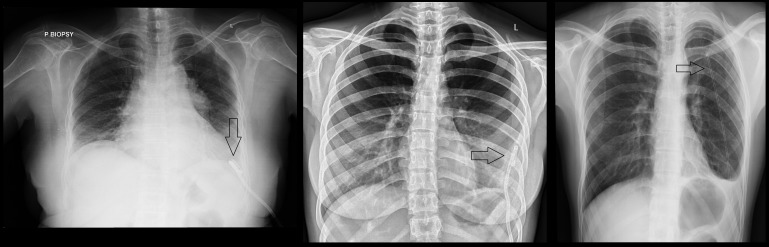
The figure shows the catheter in place in a patient with malignant pleural effusion (left), empyema (center) and primary spontaneous pneumothorax (right)

For the straight drain, a preloaded 12-gauge needle was used to puncture the site under ultrasound guidance. Once in the pleural space, the catheter was fed through the needle and the needle eventually removed. The amount of air aspirated was measured. In case of pigtail catheter, under fluoroscopic and or ultrasound guidance, an 18-gauge needle was used to puncture the selected site. The amount of fluid aspirated was measured; color was noted and sent for routine analysis along with any other test that aided in the confirmation of the suspected diagnosis, if not available before. A guidewire that was passed through the needle and the pigtail catheter was then inserted. The guidewire was removed once the catheter was in place. Figure 1 shows radiographs of patients after the insertion of the catheter.

The catheter was sutured to the skin using silk. A Heimlich valve, in the case of a pneumothorax, or a drainage bag, in the case of a pleural effusion or empyema, was attached to end of the catheter. A post-procedure chest X-ray was done to ensure correct positioning of the IPC, to rule out any procedural complication, and to compare the change/resolution of the radiological appearance of pleural pathology with pre-procedural chest X-ray.

The patients' vital signs were again monitored for a few minutes to ensure stability. The patient and his or her relatives were counseled regarding care of the IPC and the attached Heimlich valve or drainage bag. The patient was subsequently referred to the pulmonary clinic for final instructions regarding maintaining a daily record of fluid drainage or any other queries about home management before leaving the hospital. They were advised to follow-up for subsequent decisions regarding drain removal and other interventions like chemical pleurodesis in the pulmonary clinic.

Any complications encountered by the patient or observed by the pulmonologist in follow-up visits were noted and managed in conjunction with interventional radiology or oncology if required.

Pleurodesis was performed in the pulmonary clinic when daily fluid output was less than 100 cc (cubic centimeters) on three consecutive days with no or minimal residual effusion on chest X-ray in case of recurrent malignant effusions or complete lung expansion in case of pneumothorax with minimal or no air leak. We used sterilized 3 to 5 grams talc slurry instilled in the pulmonary clinic under aseptic conditions. In order to minimize pain, the talc slurry was mixed with 10-20 cc of 2% lignocaine. Subsequent pain was managed with oral paracetamol and tramadol when required. In case of pneumothorax, the IPC was removed the same day after instillation of talc with a follow-up chest X-ray, while patients with malignant effusions were asked to report back after two days for IPC removal and follow-up chest X-ray.

Data on quality of life were obtained retrospectively by asking patients, or their close relatives in case the patient had died, in a telephonic interview. They were asked regarding the activities they were able to perform with the catheter in place and for any limitations of their usual daily life activities.

Operational definitions

On follow-up, patients were assessed both clinically as well as radiologically. ‘Complete clinical resolution’ was labeled when the patient’s symptoms, i.e. shortness of breath and fever, were resolved and fluid drainage had decreased to 100 cc or less in 24 hours. In case of absence of any of these criteria, ‘partial resolution’ was labeled. If there was no improvement in symptoms, ‘no resolution’ was labeled.

‘Complete radiological resolution’ was labeled when the patient’s radiographs returned to normal. If radiographs still showed some signs of the pathology, but there was a significant improvement in the gross appearance of the radiographs, the label of ‘partial radiological resolution’ was used. If there was no improvement or worsening of the radiological appearance, the patient was labeled as having ‘no radiological resolution’.

In patients in whom partial or complete clinical resolution had occurred, recurrence was considered when the patients developed the same symptoms, shortness of breath with or without fever, accompanied with worsening of the radiological appearance when compared with the radiographs at the time when the disease process was labeled as having been clinically resolved.

Success was defined as clinical resolution, either complete or partial, regardless of the radiological outcome.

Statistical analyses

The data collected were entered and analyzed using IBM's Statistical Package for Social Sciences (SPSS) (IBM Corp., Armonk, NY), version 24. Patient identifiers were removed once all the data had been entered and thoroughly reviewed to rule out any mistakes in the data entry process. Descriptive statistics were used to present the patient characteristics, any comorbidities, pulmonary and pleural pathologies, catheter size and its type, days to catheter removal, clinical and radiological resolution, any complications, and the need for alternative management. Continuous data are reported with the mean and standard deviations where required.

## Results

**Figure 2:**
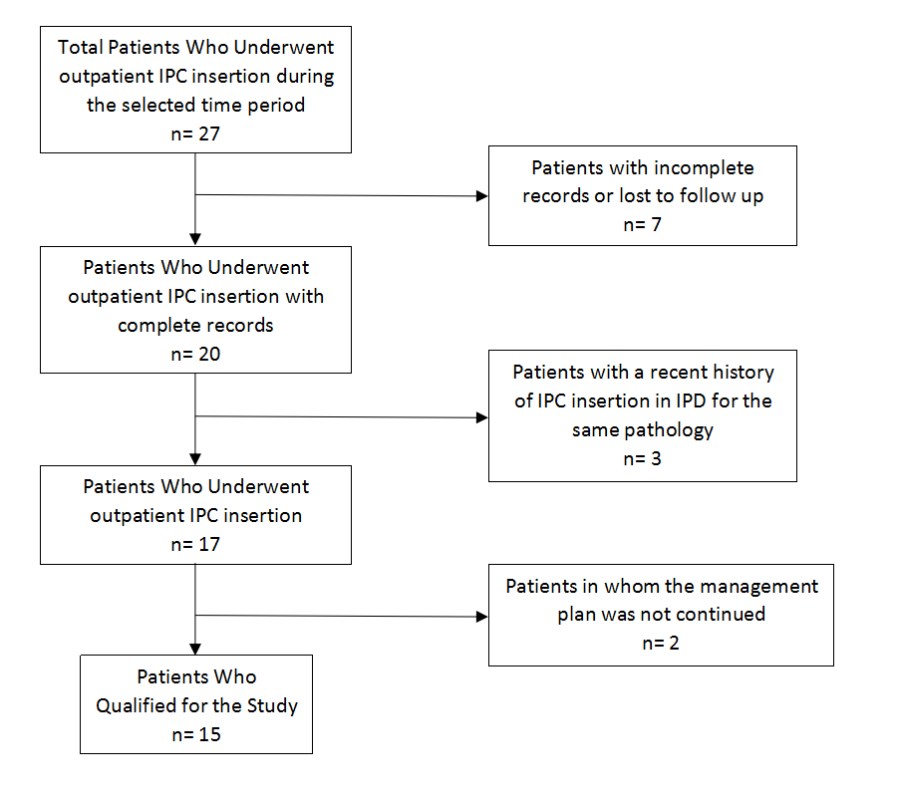
Outline of the patient selection criteria for the study IPD = inpatient department, IPC = indwelling plueral catheter

A total of 27 patients were enrolled between January 1, 2015 through December 31, 2016 who underwent IPC insertion on an outpatient or ambulatory basis. Twelve patients were excluded due to reasons mentioned in Figure 2; thus, 15 patients qualified for our study.

All patients were South Asian in origin with a mean age of 55.2 ± 16.3 years. There were seven (46.7%) males and eight (53.3%) females. Seven (46.7%) patients were diagnosed with malignant pleural effusion. The details about various causes of pleural effusions, other pleural pathologies, comorbidities, days to the removal of the catheter, and size and type of catheter inserted are mentioned in Table 1.

**Table 1:** Individual Patient Demographics, Diagnoses and Type of Catheters Used yrs = years, F = female, M = male, Fr = French, DM = diabetes mellitus, HTN = hypertension, CKD = chronic kidney disease

	Malignant Pleural Effusions						
	Age (yrs)	Sex	Diagnosis	Comorbidities	Catheter Type	Catheter Size	Days to Removal
1	70	F	Breast Carcinoma	None	Pigtail catheter	8 Fr	28
2	40	F	Breast Carcinoma	None	Pigtail catheter	8 Fr	10
3	69	F	Ovarian Carcinoma	None	Pigtail catheter	12 Fr	14
4	56	F	Pleomorphic Sarcoma (Chest Wall)	DM, HTN	Pigtail catheter	12 Fr	8
5	80	M	Leiomyosarcoma	DM	Pigtail catheter	12 Fr	11
6	60	F	Pulmonary Adenocarcinoma	None	Straight drain	10 Fr	46
7	55	F	Mesothelioma	None	Pigtail catheter	12 Fr	11
	Non-malignant Pleural Effusions						
	Age (yrs)	Sex	Comorbidities		Catheter Type	Catheter Size	Days to Removal
8	68	F	DM, HTN, CKD		Straight drain	8 Fr	30
9	68	M	CKD		Pigtail catheter	8 Fr	10
10	61	M	Limbic Vasculitis		Pigtail catheter	12 Fr	12
	Empyema						
	Age (yrs)	Sex	Comorbidities		Catheter Type	Catheter Size	Days to Removal
11	56	M	None		Pigtail catheter	10 Fr	5
12	58	M	None		Pigtail catheter	12 Fr	11
13	25	F	None		Pigtail catheter	10 Fr	6
	Primary Spontaneous Pneumothorax						
	Age (yrs)	Sex	Comorbidities		Catheter Type	Catheter Size	Days to Removal
14	30	M	None		Straight drain	8 Fr	7
15	32	M	None		Straight drain	8 Fr	6

The mean number of days to catheter removal in all patients was 15 ± 11.4 days with a minimum of five days and a maximum number of 46 days. Of the seven patients with malignant pleural effusions, three (42.9%) had a complete clinical resolution, three (42.9%) had partial clinical resolution and one (14.2%) had no clinical resolution. One patient (14.2%) had a complete radiological resolution, two (28.6%) had partial radiological resolution, and four (57.1%) had no radiological resolution. Of the three patients with nonmalignant pleural effusion, two (66.7%) had complete clinical resolution and one (33.3%) had no clinical resolution, while two (66.7%) had partial radiological resolution and one (33.3%) had no radiological resolution. Of the three patients with empyema, all three had complete clinical resolution; but only partial radiological resolution was noted. Both (100%) patients with PSP (primary spontaneous pneumothorax) had complete clinical, as well as, radiological resolution. Recurrence was noted in only two patients (13.3%) who had a diagnosis of malignant pleural effusion making up 28.6% of the patients in that category. Another patient returned with complaints of shortness of breath, which was diagnosed as a malignant pleural effusion on the contralateral side.

One patient (6.7%), comprising 14.2% of the malignant pleural effusion category, presented to the emergency room (ER) with complaints of no drain output and the problem was subsequently resolved. None of the patients required hospital admissions due to issues with the concerned procedure. One patient on hemodialysis from the nonmalignant pleural effusion category presented to the ER with hyperkalemia and was subsequently admitted for dialysis. The same patient (6.7%) had a standard large-bore chest tube placed and surgery was done due to the failure of clinical and radiological resolution, even after 30 days.

In terms of complications, one patient (6.7%) reported significant pain after the procedure. Another patient (6.7%) had a displaced catheter, while a third patient (6.7%) had kinking of the catheter. None of the patients' procedures were complicated with fever or pneumothorax.

When asked about the quality of life subsequently, one patient (6.7%) reported a 40% limitation in his daily life activities, while the other 14 patients (93.3%) did not report any decrease in their daily life activities.

Three patients with malignant pleural effusions expired after pleurodesis. The mean number of days to expiry after the procedure was 56 ± 61.9 days, with the minimum of 13 and maximum of 127 days. None of the deaths were due to complications related to the pleural pathologies being managed. Table 2 describes the outcomes for individual patients.

**Table 2:** Outcome of Intervention for Individual Patients ER = emergency room, QoL = quality of life

	Malignant Pleural Effusions							
	Clinical Resolution	Radiological Resolution	ER Visits	Chest tube	Surgery	Complications	Recurrence	Expiry (days)
1	Partial	None	0	No	No	None	No	28
2	Complete	Complete	0	No	No	None	No	-
3	Complete	Partial	0	No	No	None	Yes	-
4	None	None	1	No	No	None	No	-
5	Complete	None	0	No	No	None	No	13
6	Partial	Partial	0	No	No	None	No	127
7	Complete	None	0	No	No	None	Yes	-
	Non-malignant Pleural Effusions							
	Clinical Resolution	Radiologic Resolution	ER Visits	Chest tube	Surgery	Complications	Recurrence	
8	None	None	1	Yes	Yes	Kinking	No	
9	Complete	Partial	0	No	No	None	No	
10	Complete	Partial	0	No	No	Displacement	No	
	Empyema							
	Clinical Resolution	Radiologic Resolution	ER Visits	Chest tube	Surgery	Complications	Recurrence	
11	Complete	Partial	0	No	No	None	No	
12	Complete	Partial	0	No	No	None	No	
13	Complete	Partial	0	No	No	None	No	
	Primary Spontaneous Pneumothorax							
	Clinical Resolution	Radiologic Resolution	ER Visits	Chest tube	Surgery	Complications	Recurrence	
14	Complete	Complete	0	No	No	Decrease in QoL	No	
15	Complete	Complete	0	No	No	Pain	No	

## Discussion

We were able to manage several pleural pathologies requiring intervention on an outpatient basis with the help of small-bore indwelling catheters. Malignant pleural effusions, primary spontaneous pneumothorax, and empyema were the common causes seen in our case series. Patients tolerated these procedures well and were able to self-manage daily drainage of collection bag with some help from their family members. None of our patients were admitted, and the decisions about insertion and removal were taken and implemented on the outpatient basis.

Evidence of malignant pleural effusion (MPE) heralds stage-4 cancers, regardless of the type. The median survival of patients with MPE is three months [13]. With palliative management, the success rate has been reported around 75% at one month and more than 50% by six months, along with the reported complications [16]. Among other goals of management, improving quality of life of the patients is of the utmost importance. Avoiding admission to the hospital, a decrease in hospital stays, and early removal of tubes are considered a constituent part of the quality of life. In the last couple of decades, these factors have been the focal points of clinicians in helping progress in the management of MPE.

In 1994, Villanueva, et al. compared short-term and long-term thoracoscopic drainage before pleurodesis and demonstrated that there was no significant difference in the outcomes [17]. Subsequent studies by Patz, et al. in 1996 [18], Hsu, et al. in 1998 [19] and Marom, et al. in 1999 [20] suggested the use of small-bore catheters for drainage and pleurodesis as a comparable alternative to previously used large-bore chest tubes.

In 2000, Saffran, et al. described outpatient pleurodesis in MPE using pigtail catheters [21]. The patients were not admitted after the insertion of the catheters contrary to common practice and further management was done on an ambulatory basis unless indicated otherwise. Six out of eight patients completing the procedure reported symptomatic improvement. In 2004, Musani, et al. reported complete or partially successful pleurodesis and symptomatic improvement in 11 (58%) of 19 patients using a similar ambulatory procedure [15].

In 2003, Spiegler, et al. reported the use of rapid pleurodesis in which they did not wait for the drain output to decrease, and carried out pleurodesis within one to two days of the catheter tube insertion [22]. They reported complete response in 14 (48%) of 27 patients and a partial response in nine (31%) patients. Within the same study, two patients underwent ambulatory pleurodesis, only one of which had a partial response. While the ambulatory pleurodesis technique was similar to the one that we used, we did wait for the drain output to decrease to 100 cc or less before we proceeded with pleurodesis.

In 2011, Bediwy and Amer described the use of an ambulatory technique for multiple pleural etiologies [10]. However, they described only short-term, i.e. within 72 hours of the removal of the pigtail catheter, success. Our study focused mainly on long-term success. With a success rate of 85.7% (42.9% complete and 42.9% partial) of MPE, 66.7% of nonmalignant pleural effusions, 100% of empyema, and 100% of PSP and minimal complications, our study showed the effective use of this technique for various pleural pathologies with particular emphasis on MPE due to its tiring nature for both patients, as well as health care professionals.

Though radiological resolution was less successful in MPE (42.9% (14.2% complete and 28.6% partial)), palliation was our main focus, hence clinical resolution. In nonmalignant effusions, the radiological resolution (partial) was better at 66.7%. Similarly, in empyema, the radiological resolution (partial) was 100% at the time of follow-up. This might be explained by the fact that it takes significantly more time in such patients for imaging to return to normal. Since the patients did not have any recurrence, imaging was not followed-up after the evidence of clinical resolution and improving radiology. In contrast, however, PSP, where the radiological improvement may be noted earlier, all the patients achieved complete radiological resolution.

The use of this technique is of particular importance, especially in developing countries similar to ours. In developed welfare states, the burden of health care is decreased with cost effective solutions such as the outpatient use of IPC. In resource constrained countries, such strategies help in reducing the financial burden on patients and their families, where the cost is mostly borne by them. A cost effective solution, such as this, would allow more patients in our setup to gain access to palliation in case of malignant pleural effusion, and definitive treatment in case of the curable pleural pathologies described.

The small sample size of a highly select population is a significant limitation in our study. Moreover, lack of direct comparison with conventional treatment modalities, i.e large-bore chest tubes, thoracoscopic decortication/pleurodesis, is another limitation. Previous studies indicate fever and pneumothorax to be the most reported complications after pleural catheter insertions. Patients in our study experienced neither of these. The use of ultrasound-guided catheter insertion might account for zero pneumothoraces in our cohort. The most significant complication in our study was pain (in 6.7% of the patients). This incidence was much lower than the reported 20-45% in some studies [10, 13, 22].

Even though we did not include a formal cost analysis in our study, we expect that the cost will be much less in comparison to inpatient catheter insertions, avoiding hospital stays and surgical interventions requiring general anesthesia, operation theater services, and at times, thoracic surgical expertise.

## Conclusions

Ambulatory, small-bore, pleural catheter insertion is a feasible management option in several pleural pathologies. It is easily tolerated, can be managed at home with an improved quality of life, and is potentially less costly in resource constrained environments. Further studies can be designed to include a wider population of patients with pathologies like secondary spontaneous pneumothorax, non-malignant effusions requiring pleurodesis, and complicated empyemas requiring fibrinolysis. It will also be of interest to study its use for palliation in patients with an expected survival time of fewer than six months in our population.
